# Castleman’s Disease and Anticonvulsant Therapy: A Case of 52-Year-Old Female

**DOI:** 10.7759/cureus.20480

**Published:** 2021-12-17

**Authors:** Sarah H Alobud, Fatimah M Bukhamseen, Tariq M Hashim, Omran Al Dandan, Munir A ALrefaee

**Affiliations:** 1 Internal Medicine, King Fahad University Hospital, Al Khobar, SAU; 2 Pathology, King Fahad University Hospital, Al Khobar, SAU; 3 Radiology, King Fahad University Hospital, Al Khobar, SAU

**Keywords:** multicentric castleman's disease, giant benign lymphoma, lymphadenopathy, lymphoproliferative disorder, angiofollicular lymph node hyperplasia

## Abstract

Castleman’s disease is a disorder of the lymph nodes that encompasses heterogeneous clinical conditions and can be classified into two main types - hyaline vascular and plasma cell. The affected age group ranges widely from two to 80 years old at the time of diagnosis, with a variable clinical presentation. The etiology of Castleman’s disease is not yet well-understood; however, a number of factors have been linked to its pathogenesis including certain cytokines, viral infections, autoimmunity, immunodeficiency, chronic inflammation, and Kaposi sarcoma. In this study, we present a case of a 52-year-old Saudi female with a history of pulmonary embolism and deep venous thrombosis, who was then found to have mild splenomegaly, mediastinal, bilateral hilar, supraclavicular, paraaortic, and right axillary lymphadenopathy on CT scan, to be later diagnosed as a multicentric Castleman’s disease (MCD). Moreover, the clinical picture, pathogenesis, clinical and histological variants, as well as the treatment options of MCD are discussed.

## Introduction

Castleman’s disease is a life-threatening lymphoproliferative disorder that encompasses a heterogeneous group of clinicopathological conditions. An incidence of 4300 to 5200 per year is estimated in the United States [1], with the age of diagnosis widely ranging from two to 80 years [2]. The disease was first reported in 1954 by Dr. Benjamin Castleman after a 40-year-old man presented with a mediastinal mass with a histopathological diagnosis of lymphoid tissue hyperplasia and hyalinized germinal centers [3]. Subsequently, several cases were documented showing two main subcategories of the disease, each with different distinguishable features. The first was the hyaline vascular type with follicular lymphoid hyalinization and angiogenesis, while the other was the plasma cell type with prominent plasma cell proliferation [4]. All the cases were presented with solitary lesions, mainly in the mediastinum. In 1983, a case series was published describing a different entity of the disease. Fifteen cases with involvement of multiple lymph nodes showed features of both vascular hyalinization and plasma cell proliferation, leading to the establishment of multicentric Castleman’s disease (MCD) diagnosis [5].

In 1995, an etiological agent was identified by Soulier et al. after studying 14 HIV-associated cases, and all tested positive for human herpesvirus 8 (HHV-8), also known as Kaposi's sarcoma-associated herpesvirus. Furthermore, HHV-8 was identified in 40% of HIV-negative cases [6]. More data collected corroborated this finding, as HHV-8 was found to be associated with around two-thirds of MCD cases, while the remaining third is classified as HHV-8 negative/idiopathic multicentric Castleman’s disease (iMCD) [2]. The pathogenesis of MCD is not well elucidated; nonetheless, an Interleukin-6 (IL-6)-derived neoplastic process has been described and was linked to the systemic manifestation of the disease [7]. Interestingly, HHV-8 encodes a viral analog of IL-6, possessing both hematopoietic and angiogenic effects [7]. However, this theory was not consistent with studies of iMCD pathogenesis. Various mechanisms have been proposed including autoimmune, autoinflammatory, neoplastic, and infectious [1].

iMCD constitutes a diagnostic challenge provided that it’s primarily a diagnosis of exclusion [8]. Moreover, iMCD is considered a rare condition, with very limited studies published in regards to its etiology. In this study, we present a case of iMCD, initially suspected to be a lymphoma case, with a history of long-term use of the antiepileptic medication, carbamazepine.

## Case presentation

A 52-year-old Saudi female, a known case of epilepsy on carbamazepine, with a history of pulmonary embolism (PE) and deep venous thrombosis (DVT) presented to the emergency department (ED) of King Fahad University Hospital in Al Khobar city, complaining of cough for three days. She was in her usual state of health, until five days prior to her presentation, when she developed a cough that was productive in nature, with a small amount of yellow sputum. She also reported a history of shortness of breath (SOB) which started suddenly and did not improve until she received a nebulized bronchodilator, and was discharged from the ED on antibiotics. Her symptoms were associated with pleuritic pain mainly in the right lower part of the chest, loss of appetite, unintentional significant weight loss, and two episodes of vomiting. However, she denied any history of hemoptysis, fever, night sweats, chills, rigors, abdominal pain, change in bowel motion, hematuria, or dysuria.

At the age of 20 years, the patient was diagnosed with epilepsy (recurrent generalized tonic-clonic seizures) and was on carbamazepine 400 mg once daily for 32 years. She had her last fit three weeks prior to her symptoms. Her condition was aggravated by sleep deprivation and mostly occurred at night (nocturnal seizures). Furthermore, the patient was admitted in 2012 as a case of DVT with a subsequent massive PE for which she was kept on warfarin.

The physical examination was unremarkable except for enlarged bilateral axillary and inguinal lymph nodes. The patient’s laboratory investigation revealed pancytopenia. Additionally, a chest x-ray was done which showed bilateral hilar lymphadenopathy and right lower region consolidation, suggesting the diagnosis of community-acquired pneumonia (CAP). Upon the previous findings, the patient was admitted and started on empirical antibiotics. During this hospital stay, she underwent a number of investigations in order to establish the underlying pathology behind the previously mentioned findings. In addition to a pan-computed tomography (CT), pulmonary function tests (PFTs) were ordered. The findings of both investigations were consistent with the provisional diagnosis of suppurative lung disease. PFTs revealed a restrictive pattern, while the pan-CT showed bilateral bronchiectasis, right-sided pneumonia, and bilateral pleural effusion. However, additional findings on pan-CT had raised the suspicion of lymphoma, including mild splenomegaly, mediastinal and bilateral hilar, supraclavicular, and paraaortic lymphadenopathy. Moreover, during the course of this admission, the patient had developed another DVT, in which she was started on low molecular weight heparin then bridged to warfarin.

**Figure 1:**
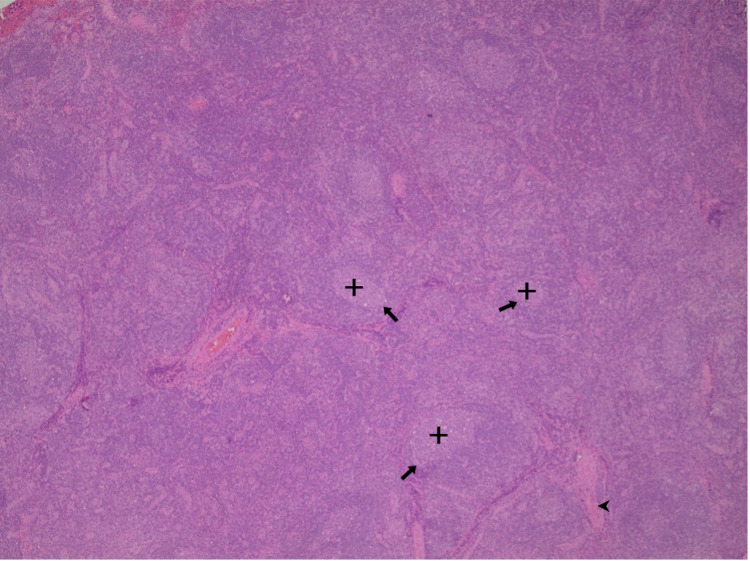
Variable sized lymphoid follicles with involuted germinal centers Regressive transformation and increased vascular proliferation show hyaline change (H&E x4). (Arrowhead - hyaline changes; arrows - FDCs; cross - germinal centers) FDCs: follicular dendritic cells

**Figure 2:**
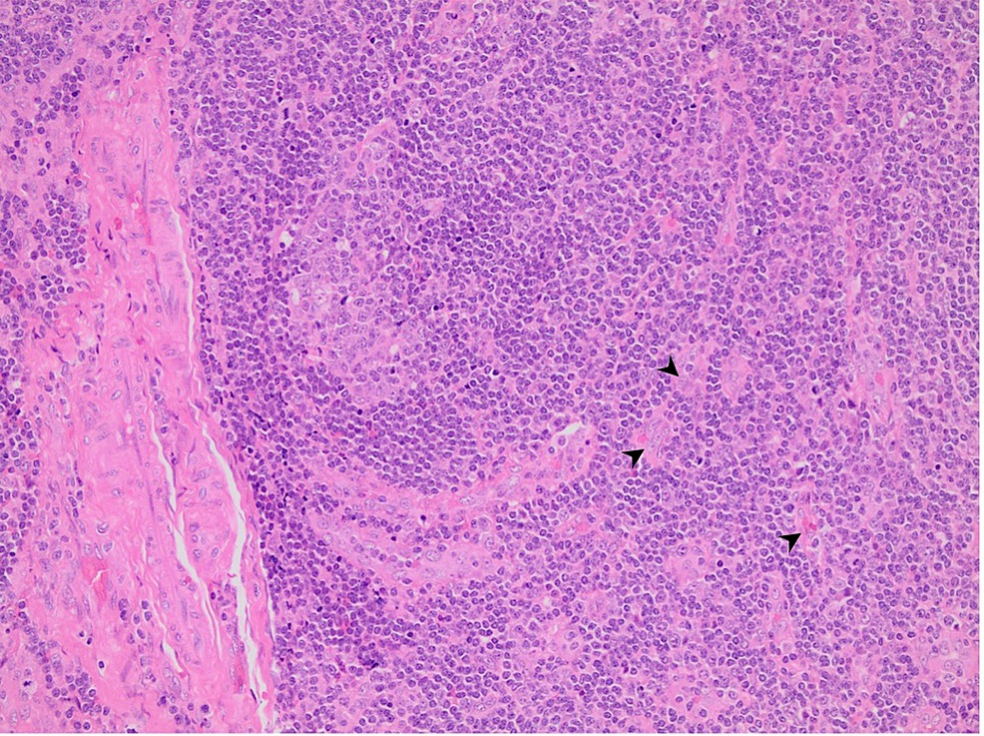
Increased vascular proliferation showing hyaline change (H&E x20). (Arrowheads - hyaline changes)

**Figure 3:**
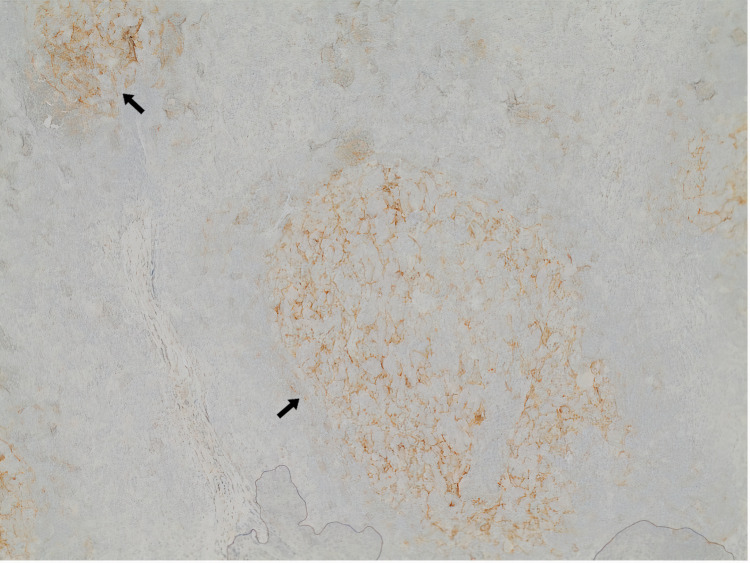
Follicular dendritic cells The image shows constant distribution within germinal centers, with intense positivity and accentuated expansion towards the hyperplastic mantle zone (CD21 x10). (Arrows - FDCs) CD: cluster of differentiation; FDCs: follicular dendritic cells

**Figure 4:**
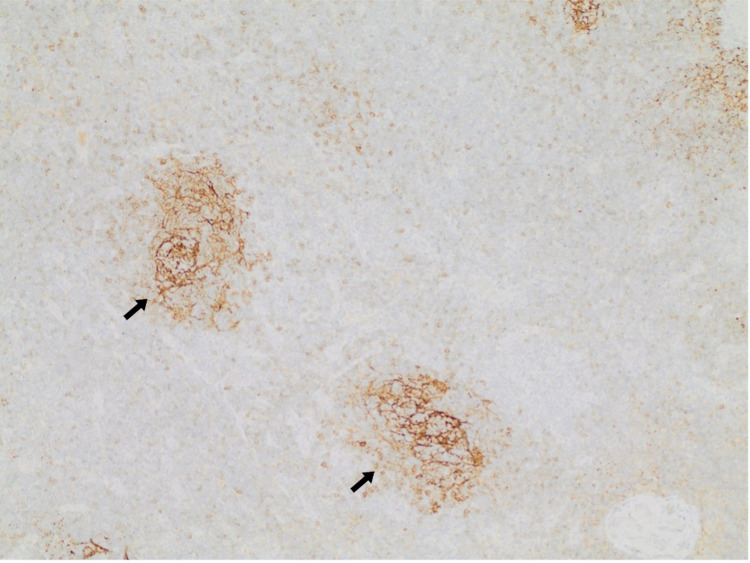
CD23 highlights follicular dendritic cell hyperplasia (FDCs) (CD23 x10). (Arrows - FDCs) CD: cluster of differentiation; FDCs: follicular dendritic cells

After two weeks from admission, an excisional biopsy of a single enlarged right axillary lymph node was taken. On histopathology, the lymph node was hyperplastic with features directing toward Castleman’s disease of a lymphocytic variant of the hyaline vascular type. Furthermore, leukocyte-common antigen (LCA), cluster of differentiation (CD)3, CD5, CD20, CD21, CD15, CD30, cyclin D1, Bc1-2, Bc1-6, Epstein-Barr virus (EBV), and reticulin staining were all suggestive of CD as illustrated in Figures 1-4. 

**Figure 5:**
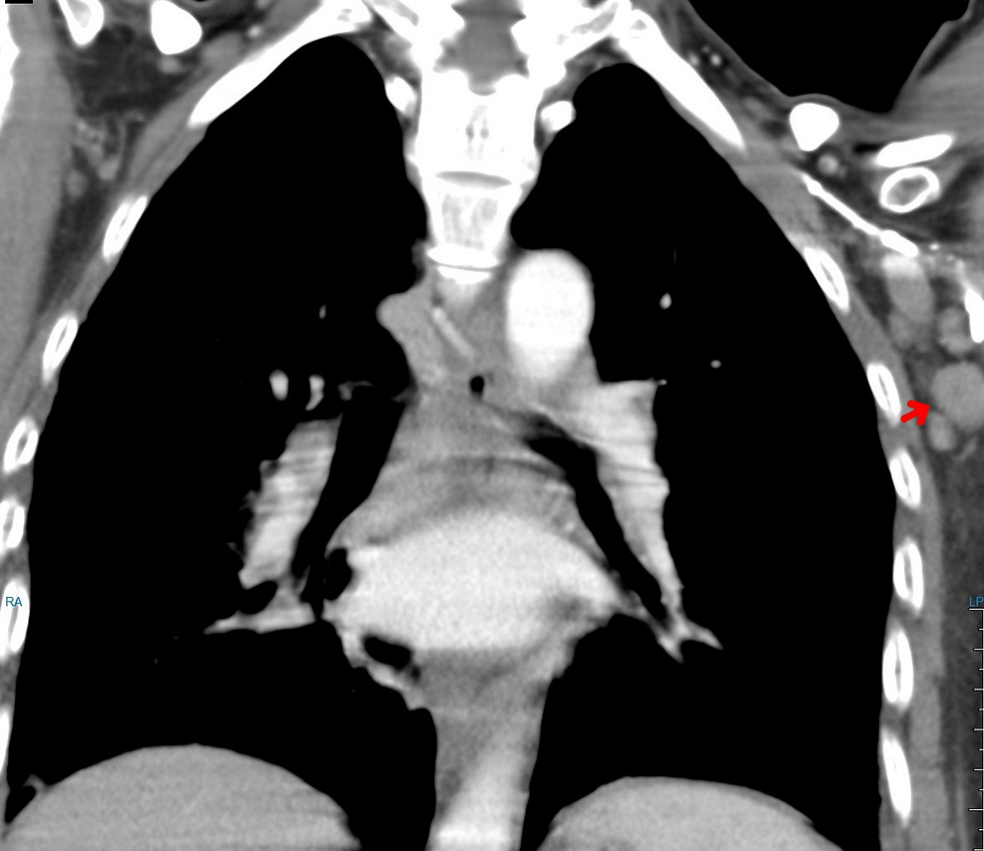
Chest CT (February 17, 2016) showing enlarged left axillary lymph node of 2.5 cm

**Figure 6:**
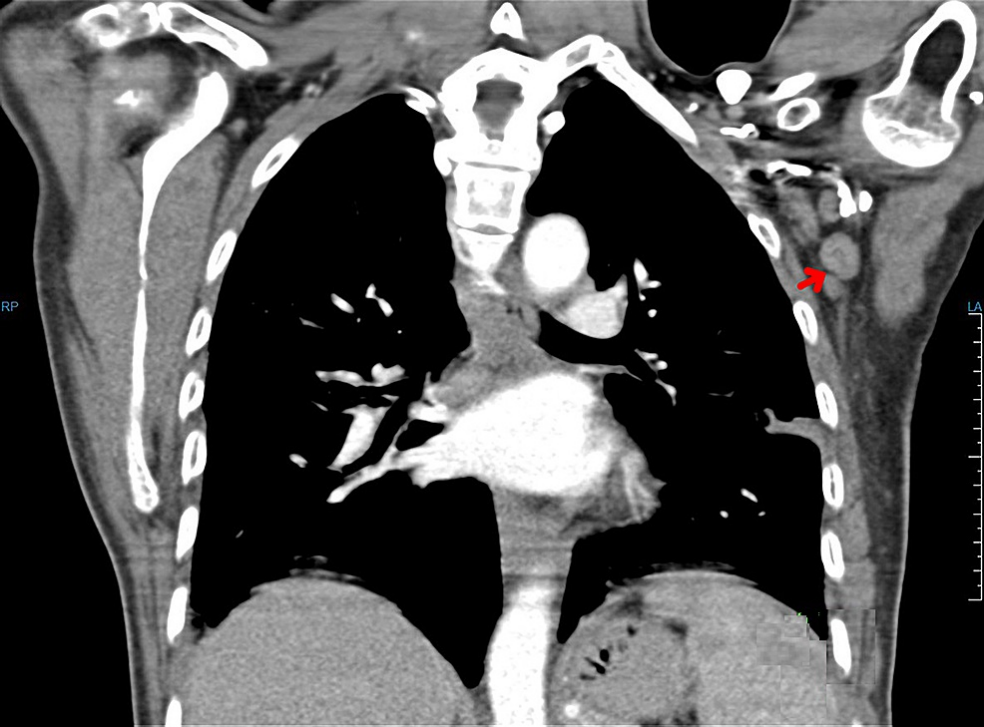
Chest CT (May 22, 2016) showing enlarged left axillary lymph node of 1.3 cm

Based on these findings, a shared decision was made to stop carbamazepine, and repeat the CT scan to reassess the size of lymph nodes. It revealed an interval regression in the size of the left axillary lymph nodes as illustrated in Figure 5 and Figure 6. The patient, therefore, was discharged and instructed to follow-up with the oncology department, in which she elected to receive biological therapy with rituximab at first. She only minimally responded to this type of therapy; subsequently, management proceeded with six cycles of chemotherapy rituximab, cyclophosphamide, doxorubicin, vincristine, and prednisone (R-CHOP) regimen and achieved full remission. 

## Discussion

Castleman’s disease, angiofollicular lymph node hyperplasia, and Castleman tumor are all synonyms that describe a pathology of the proliferation of lymph nodes and lymphoid tissue. It has been classified into unicentric and multicentric diseases. Additionally, subsequent variants have been documented based on the histopathological findings of lymph nodes’ biopsies [9]. The first subtype is called hyaline variant Castleman’s disease, which is characterized by the presence of small follicles of hyaline-vascular origin along with capillary proliferation. The second subtype is called the plasma cell variant Castleman’s disease, in which the lymphoid follicles are larger and separated by plasma cells that are arranged in sheets. The third variant is a mixed form of the previous two types, in which it contains both hyaline features and plasma cells. The fourth and last variant is called the plasmablastic Castleman’s disease, which is more likely to be associated with HHV-8 infection, which affects people of low or no immunity, and has the potential of progressing into plasmablastic lymphoma [10].

The pathophysiology and etiology of Castleman’s disease are not yet well-understood; however, there are a number of factors that have been debated to contribute to its pathogenesis. This includes IL-6 cytokine, viral infections, and vascular endothelial growth factor cytokine (VEGF). IL-6 cytokine plays a major role in the production of B cell lymphocytes and has been significantly correlated to the development of a number of malignancies including lymphoma and multiple myeloma. Additionally, it has been noticed to be elevated in patients with Castleman’s disease, which was hypothesized to be secondary to the downregulation of IL-6 receptor signaling pathway, or to the dysregulation of its production. Another possible explanation is the presence of HHV-8 infected cells, as found in subjects with MCD. These cells produce an analog to the endogenous IL-6 cytokine, resulting in its increased serum levels and function, as well as the level of VEGF, which is important for angiogenesis [10]. Furthermore, MCD and Kaposi sarcoma (KS) have been known to coexist, as KS is seen in 75% of patients with MCD who tested positive for HIV, and in 13% of HIV-negative patients. The literature clearly showed that HHV-8 viral infection is significantly correlated with the development of MCD in HIV-positive patients and is considered a major risk factor [11]. Definite causation however is still challenging due to the low incidence of Castleman’s disease [10,11]. Other suggested etiological factors include autoimmunity, immunodeficiency, chronic inflammatory response, environmental triggers, and Epstein-Barr virus infection [9].

Castleman’s disease must be differentiated from other pathologies that can result in lymphoid hyperplasia and mimic lymphoma. Interestingly, anticonvulsant therapy, particularly phenytoin and carbamazepine, can result in lymph node reactivity and proliferation, leading to anticonvulsant hypersensitivity syndrome (AHS). The histopathological findings of lymph node biopsy in AHS are distinct from the features seen with Castleman’s disease, with no hyalinization, hyperplastic follicles, or interfollicular vessels proliferation [12]. The patient, in this case, was found to have MCD proven by pathology report of axillary lymph node (LN) biopsy specimen. Nonetheless, the fact that she was kept on carbamazepine therapy for 32 years has raised a question about whether carbamazepine and MCD have some association, similar to that seen between it and AHS. Moreover, as the data available in the literature regarding Castleman’s disease is lacking due to its low incidence, none of the existing literature has addressed this questioned issue. In most diagnosed Castleman’s disease cases, a single definitive etiology cannot be made; thus conducting more studies in the future is crucial in order to investigate the association of Castleman’s disease and anticonvulsant therapy, particularly; carbamazepine. 

The clinical picture of MCD tends to be severe in nature as the majority of patients present with high-grade fever, fatigue, weakness, significant weight loss, night sweat, and anorexia. Moreover, in the advanced stages of the disease, patients might present with ascites, pleural effusion, pericardial effusion, and malignant rash, or it can occur in the context of polyneuropathy, organomegaly, endocrinopathy, monoclonal gammopathy, and skin changes (POEMS) syndrome [10].

Thrombogenic events have also been reported in some cases of Castleman’s disease as a presenting complaint. This complication is commonly reported in the vessels of the lower limbs, or the coronary arteries, and might be attributed to a number of factors. The main and most implicated factor is the mass effect exerted by the enlarged lymph nodes over deep veins. Another factor is the increased levels of IL-6 cytokine, which acts as a procoagulant factor, promoting thrombosis and chronic inflammation [13]. Other mechanisms of thrombosis in the context of Castleman’s disease include autoimmune deregulation, as seen in cases of Behcet disease, nephrotic syndrome, and antiphospholipid syndrome, or when the disease coexists with POEMS syndrome [14]. Similarly, the patient, in this case, had a history of multiple thromboembolic events as mentioned earlier, in which they were later thought to be brought by the hypercoagulable state attributed by MCD.

The clinical approach and diagnosis of Castleman’s disease must involve meticulous history taking, physical examination, laboratory workup, and imaging modalities. The definite diagnosis however cannot be made without an excisional biopsy of the affected lymph nodes, which shows the characteristic plasma cells, or follicles of hyaline-vascular origin [11]. When investigated with laboratory tests, the results might show severe anemia of chronic disease or hemolytic type, thrombocytopenia, hypoalbuminemia, elevated inflammatory markers mainly erythrocyte sedimentation rate (ESR), elevated levels of immunoglobulins, and impaired liver function test [10]. 

Treatment options of Castleman’s disease include chemotherapy, radiotherapy, immunomodulatory agents, and biologics (monoclonal antibodies) [9,11]. The selection of therapy depends on the severity of the disease, in addition to whether MCD is isolated or occurring within the context of HHV-8, HIV, or POEMS syndrome. Moreover, treatment of mild forms of MCD at which patients are asymptomatic may only require the use of indefinite siltuximab therapy in order to achieve and maintain a full remission. The use of combined rituximab and glucocorticoids therapy, with or without immune-modulator agents, might be indicated for more clinically evident disease, at which patients are minimally symptomatic, and show no signs of organ failure. On the other hand, patients with more clinically aggressive forms of MCD who show evidence of progressive organ dysfunction must be treated with chemotherapy, in addition to the use of siltuximab. The decision about whether to treat with a single cytotoxic medication or multi-agent chemotherapy depends on the clinical status and performance of the patients and must be tailored for each case [15].

## Conclusions

This case report of a 52-year-old female known to have epilepsy and diagnosed with iMCD highlights the challenging process of establishing such a diagnosis. iMCD is mainly a diagnosis of exclusion, requiring a full assessment looking for any possible etiologies leading to lymph tissue proliferation. Furthermore, histological evaluation is a crucial step in the establishment of iMCD diagnosis distinguishing it from other pathologies like AHS. Additionally, this study discussed the different aspects of MCD in terms of presentation, diagnosis, and treatment options, in order to provide a comprehensive review for physicians to distinguish and recognize the diverse forms of the disease. It also shed light on the possible association of carbamazepine therapy and the development of MCD away from AHS, which mandates further studies in the future.
